# Association between ambient air particulate matter and human health impacts in northern Thailand

**DOI:** 10.1038/s41598-023-39930-9

**Published:** 2023-08-07

**Authors:** Titaporn Supasri, Shabbir H. Gheewala, Ronald Macatangay, Anurak Chakpor, Surat Sedpho

**Affiliations:** 1 Atmospheric Research Unit, National Astronomical Research Institute of Thailand, Chiang Mai, Thailand; 2 Energy Engineering Program, Faculty of Engineering, Chiang Mai University, Chiang Mai, Thailand; 3 The Joint Graduate School of Energy and Environment (JGSEE), King Mongkut’s University of Technology Thonburi, Bangkok, Thailand; 4 Center of Excellence on Energy Technology and Environment, Ministry of Higher Education, Science, Research and Innovation, Bangkok, Thailand; 5 Institute of Environmental Science and Meteorology, University of the Philippines Diliman, Quezon City, Philippines; 6 School of Energy and Environment, University of Phayao, Phayao, Thailand

**Keywords:** Environmental sciences, Environmental impact, Risk factors

## Abstract

Air pollution in Thailand is regarded as a serious health threat, especially in the northern region. High levels of particulate matter (PM_2.5_ and PM_10_) are strongly linked to severe health consequences and mortality. This study analyzed the relationship between exposure to ambient concentrations of PM_2.5_ and PM_10_ by using data from the Pollution Control Department of Thailand and the burden of disease due to an increase in the ambient particulate matter concentrations in northern Thailand. This study was conducted using the Life Cycle Assessment methodology considering the human health damage impact category in the ReCiPe 2016 method. The results revealed that the annual average years of life lived with disability from ambient PM_2.5_ in northern Thailand is about 41,372 years, while from PM_10_ it is about 59,064 years per 100,000 population. The number of deaths from lung cancer and cardiopulmonary diseases caused by PM_2.5_ were approximately 0.04% and 0.06% of the population of northern Thailand, respectively. Deaths due to lung cancer and cardiopulmonary diseases caused by PM_10_, on the other hand, were approximately 0.06% and 0.08%, respectively. The findings expressed the actual severity of the impact of air pollution on human health. It can provide valuable insights for organizations in setting strategies to address air pollution. Organizations can build well-informed strategies and turn them into legal plans by exploiting the study’s findings. This ensures that their efforts to tackle air pollution are successful, in accordance with regulations, and contribute to a healthier, more sustainable future guidelines on appropriate practices of air pollution act/policy linkage with climate change mitigation.

## Introduction

**Figure 1:**
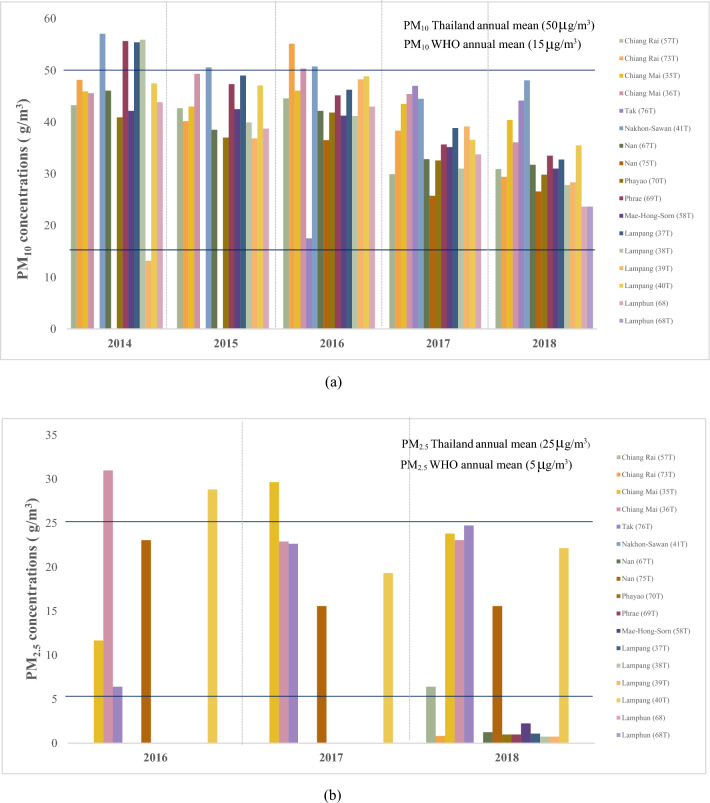
(**a**) Annual mean PM_10_ concentrations (μg/m^3^) of 16 monitoring stations in northern Thailand during 2014–2018 and (**b**) Annual mean PM_2.5_ concentrations (μg/m^3^) of 16 monitoring stations in northern Thailand during 2016–2018 (For guideline value: PM_10_ Thailand annual mean = 50 μg/m^3^, PM_2.5_ Thailand annual mean = 25 μg/m^3^, PM_10_ of World Health Organization (WHO^2^) annual mean = 15 μg/m^3^ and PM_2.5_ WHO annual mean = 5 μg/m^3^).

Air pollution is one of the world’s most critical issues with regards to health and the environment. It relates to the pollution of the environment by hazardous chemicals or biological materials. Thailand’s air pollution is regarded as one of the country’s most critical environmental issues. The significance of the problem is more severe for people living in cities, where air pollution levels are significantly greater. Power plants, manufacturing, vehicles, forest fires, agricultural burning are the major sources of air pollution. Air pollution is a chronic problem particularly in the northern region of Thailand. Farmers burn fields to clear land every year during the dry season, and there is also a higher risk of wildfires due to the vegetation type and climate. According to the Pollution Control Department (PCD^1^) of Thailand, the highest daily average of particles less than 2.5 µm in diameter (PM_2.5_) and particles less than 10 µm in diameter (PM_10_) concentrations during the haze period (January-March) in 2020 at the Mae Sai District Health Office, Chiang Rai station (73 T) was 366 and 396 μg/m^3^, respectively. In reference to the PM_2.5_ and PM_10_ guideline values in Thailand provided by the PCD, the 24 h average values should not exceed 50 and 120 μg/m^3^, respectively. The maximum daily average PM_2.5_ and PM_10_ concentrations for 10 provinces located in the north of Thailand exceeded the standard from January to March for 70 and 27 days, respectively. The annual mean PM_2.5_ and PM_10_ concentrations (μg/m^3^) of 16 monitoring stations operating in northern Thailand during 2014 to 2018 are presented in Fig. 1.

**Figure 2:**
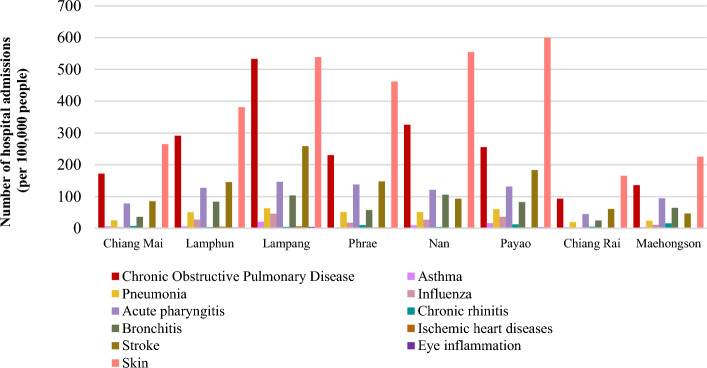
Number of hospital admissions affected by air pollution in northern Thailand in 2020.

With regards to the health impact of ambient air pollution exposure in northern Thailand, the number of hospital admissions for 11 diseases caused by air pollution, which include cardiovascular and respiratory diseases, as well as eye inflammation and skin inflammation from the Health Data Center (HDC^3^) of Ministry of Public Health in 2020 was about 588,808 cases and the incidence rate was 10,564.61 per 100,000 people. The highest number of diseases caused by air pollution were respiratory diseases equal to an incidence rate of 4124.86 per 100,000 people. This was followed by skin inflammation, eye inflammation, and cardiovascular disease at about 3191.93, 1903.81 and 1043.78 per 100,000 people, respectively. However, the data on the health effects of air pollution in the study area is limited. Figure 2 shows the number of hospital admissions affected by air pollution in the north of Thailand during January to December 2020.

Primary and secondary aerosols in the atmosphere caused by air pollution can have a significant negative influence on human health, including from respiratory symptoms to hospital admissions and mortality^2,4^. PM_2.5_ is a combination of organic and inorganic compounds. Inhaling PM_2.5_, which reaches the upper respiratory tract and lungs, causes health risks for people. Sulfur dioxide (SO_2_), nitrogen oxides (NO_x_), and other airborne pollutants among others result in the formation of secondary PM_2.5_ particles^5^. According to WHO studies, PM_2.5_ rather than coarser particulate matter is more likely to be responsible for the mortality effects of long-term exposure to air pollution. However, respiratory morbidity is linked to PM_2.5–10_.

**Figure 3:**
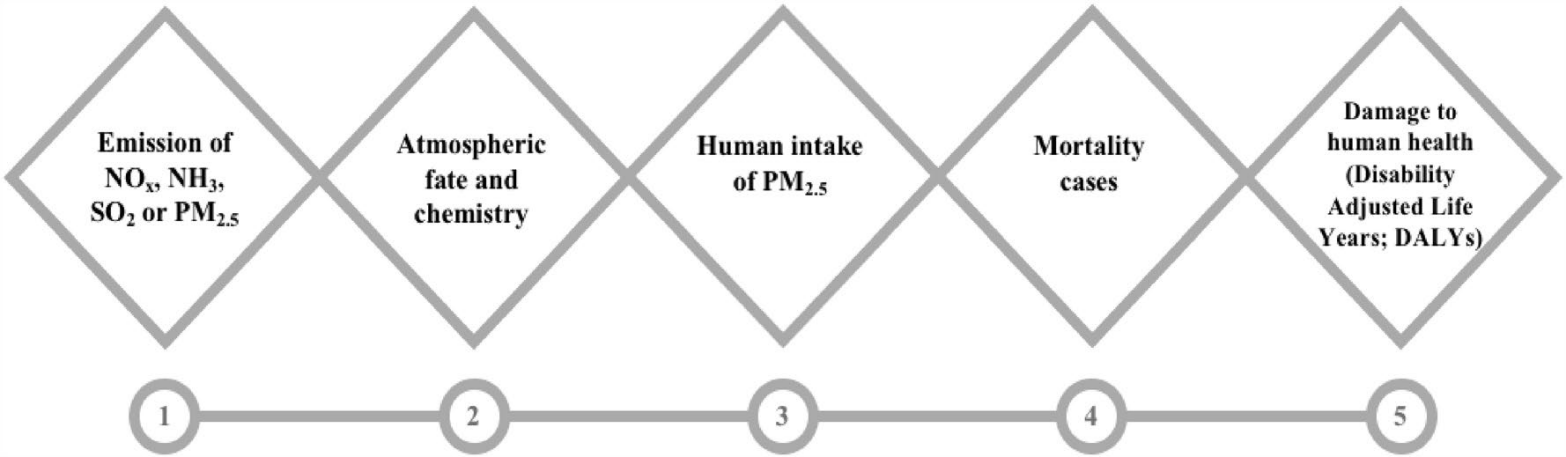
The cause-and-effect relationship of fine particulate matter emissions to human health damage^6^.

The modeling framework is divided into five parts, starting with emission to damage., shown in Fig. 3. These are: (1) emission of secondary PM_2.5_ or primary PM_2.5_; (2) atmospheric fate and chemistry; (3) human inhalation of PM_2.5_; (4) increase in the number of human deaths; (5) human health damage. To express the number of life years lost due to health problems caused by PM_2.5_ exposure., the Disability Adjusted Life Years (DALY) is used as a calculation.

Numerous studies have been published regarding air pollution and its implications on health. Vichit-Vadakan and Vajanapoom^7^ discussed the detrimental effects of air pollution in Thailand, emphasizing current and future issues. In China, Kan et al.^8^ evaluated the health impact of air pollution and climate change. Matus et al.^9^ utilized an Emissions Prediction and Policy Analysis model to assess the health implications of air pollution on the Chinese economy. Guarnieri and Balmes^10^ focused on clinical studies published in the past 5 years, covering epidemiological and experimental research. They highlighted the likelihood of oxidative harm to the lungs due to air pollutants, leading to inflammation, remodeling, and increased susceptibility. The authors also addressed symptoms, policy issues, and research needs related to air pollution and asthma.

Beelen et al.^11^ examined the relationship between natural-cause mortality and long-term exposure to various air pollutants across 22 European countries. Li et al.^12^ recently investigated the effect of model resolution on mortality attributable to PM_2.5_ and its species in the United States using GEOS-Chem, a global 3D atmospheric composition model. Pinichka et al.^13^ evaluated the burden of disease attributed to ambient air pollution in Thailand using the World Health Organization’s comparative risk assessment (CRA) framework and the Global Burden of Disease study (GBD). They implemented GIS-based exposure assessments and spatial interpolation models to estimate ambient air pollutant concentrations. Pardthaisong et al.^14^ studied the resilience of Chiang Mai, Thailand, across various sectors such as academia, government, private sector, and local communities during the period of 2007 to 2016. The study highlighted the challenges faced, particularly at the community/village level, where access to higher levels of society is limited.

Hwang et al.^15^ explored the relationship between ambient NO_2_ and PM_10_ concentrations and adult breast cancer death rates and incidence using multivariable beta regression. The findings revealed a substantial positive correlation between ambient air pollution concentrations and the incidence rate of breast cancer in the South Korean female population. Jia^16^ studied the effects of an aggregated strategic plan on damages and air pollution charge fees in China, which resulted in three benefits: reduced congestion and emissions, an improved health impact index, and a decrease in the number of illegal travels. In Bangkok, Thailand, Chavanaves^17^ calculated the health and economic gains achieved compared to a business-as-usual scenario. The associated health burden was evaluated using impact characterization factors (CFs) developed for different spatial situations in Thailand.

Ruchirawat et al.^18^ examined the potential health impacts of carcinogenic air pollution exposure in urban areas compared to rural areas, using a toxicological model of exposure to early biological effects. They assessed several biomarkers to evaluate the potential health risks associated with this exposure. Van Zelm et al.^19^ updated CFs for human health damage caused by PM_10_ and ozone in Europe in 2000, considering slight increases in NH3, NO_x_, SO_2_, PM_10_, and NMVOC emissions. Cohen et al.^20^ utilized accelerator-based ion beam analysis (IBA) techniques to quantify and characterize PM_2.5_ pollution for a range of elements from hydrogen to lead in Hanoi, Vietnam. Apte et al.^21^ examined global trends of intra-urban intake factors (IFs) for dispersed ground-level primary pollutant emissions across countries, regions, and cities of different sizes.

Kassomenos et al.^22^ investigated the relationship between ozone (O_3_) and PM_10_ exposure and public health, revealing a quantification of the disease burden from PM_10_ and O_3_-related mortality and morbidity using a Life Cycle Impact Assessment focused on Greece, specifically Athens. Gronlund et al.^23^ analyzed and evaluated CFs (DALY/kgPM_2.5emitted_) in US metropolitan regions, along with the results of dose–response factors, severity factors, and intake fractions. The studies discovered that the average annual health burden in the US due to PM emissions and CFs was 2.2 times higher.

Tang et al.^24^ assessed human health damage factors (DFs) using the Special Report on Emission Scenarios (SRESs) of the Intergovernmental Panel on Climate Change (IPCC). DFs included malaria, diarrhea, cardiovascular disease, starvation, coastal floods, and inland flooding. Van Zelm et al.^25^ determined regionalized CFs for human health damage from PM_2.5_ and ozone, as well as vegetation damage from ozone, using a chemical transport model. Khaniabadi et al.^26^ studied the effects of PM_10_, NO_2_, and O_3_ on health in Kermanshah City, Iran, finding that PM_10_ accounted for 62% of premature deaths, while NO_2_ and O_3_ accounted for 11% and 27% of deaths, respectively. They also found that a 10 μg/m^3^ increase in PM_10_, NO_2_, and O_3_ resulted in a relative risk (RR) of 1.066, 1.012, and 1.020, respectively.

Tang et al.^27^ estimated ozone DFs by region and investigated the impacts of long-distance migration on the DFs using a global chemical transport model (CTM). Additionally, Tang et al.^28^ employed a chemical transport model to evaluate the DFs of human health damage caused by PM_2.5_ in 10 different locations worldwide.

Considering the scarcity of health-related data, determining the specific health effects of PM_2.5_ for each area in Thailand is challenging. Additionally, some data such as RR, mortality rate (MR), years of life lost (YLL), and breathing rate (BR) for each province are only available as average data at the national level and not specific to local areas. Many studies rely on WHO reports or use regional and global values. It is crucial to have more accurate and reliable health data for Thailand to understand how severe air pollution affects human health in different contexts and air pollution sources in each province of Northern Thailand. Therefore, this study employs the Life Cycle Assessment (LCA) approach, following International Organization for Standardization (ISO)^29^ standards (ISO 14040^30^ and ISO 14,044), to investigate the health impacts of PM_2.5_ and PM_10_. The study aims to analyze the relationship between exposure to ambient concentrations of PM_2.5_ and PM_10_ using data from the Pollution Control Department (PCD), providing a quantification of the burden of disease from PM_2.5_ and PM_10_-related mortality and morbidity due to exposure in terms of Disability Adjusted Life Years or DALYs^31^.

The study is limited to assessing the impact of outdoor air pollution. This is because the concentration data used in the study only measure outdoor air pollution levels. It is worth noting that a significant portion of the population spends the majority of their time in non-air conditioned environments, such as homes, workplaces, and public spaces. Therefore, indoor pollution sources, such as cooking fumes and other pollutants, were not taken into consideration in this study.

Indoor pollution tends to disperse rapidly and does not accumulate to the same extent as outdoor pollution. As a result, the study focused specifically on the effects of outdoor air pollution on human health. The limitations related to indoor pollution sources and their potential health impacts were not addressed in this particular study.

## Methodology

### Goal and scope of this study

The goal of this study is to assess the human health damage caused by PM_2.5_ and PM_10_ in northern Thailand. The study employed the Life Cycle Assessment (LCA) methodology, specifically focusing on the human health damage impact category within the ReCiPe 2016 method^6^. The impact of particulate matter on human health is commonly measured in Disability-Adjusted Life Years (DALY). The study examines the relationship between exposure to ambient concentrations of PM_2.5_ and PM_10_, utilizing the annual mean PM concentration data from Thailand’s Pollution Control Department (PCD)^1^ during the period of 2014 to 2018. Additionally, the study aims to quantify the burden of disease associated with PM_2.5_ and PM_10,_ including mortality and morbidity resulting from exposure to these pollutants.

### Focus area

This study focuses on the northern region of Thailand, which comprises ten provinces: Chiang Rai, Mae-Hong-Son, Chiang Mai, Phayao, Lamphun, Lampang, Phrae, Nan, Tak, and Nakhon-Sawan. Most areas in northern Thailand are characterized by hilly terrain and serve as the source of several significant rivers. These hill ridges run in a north–south orientation, stretching parallel from west to east, intersected by various major valleys, particularly those near Chiang Mai, Chiang Rai, Lampang, and Nan provinces. The highest mountain in the region is Doi Inthanon, rising approximately 2595 m above mean sea level, located in Chiang Mai. Along the eastern border, adjacent to the northeastern part, lies a mountainous area known as the central highlands. Between the western mountains and the central highlands, there is a central valley in the southern portion of the region^32^. Agricultural land use in the northern region spans approximately 6,368,630 hectares, estimated to account for 40 percent of the total land use. This includes approximately 41 percent allocated to paddy fields and 32 percent dedicated to field crops^33^.

**Figure 4:**
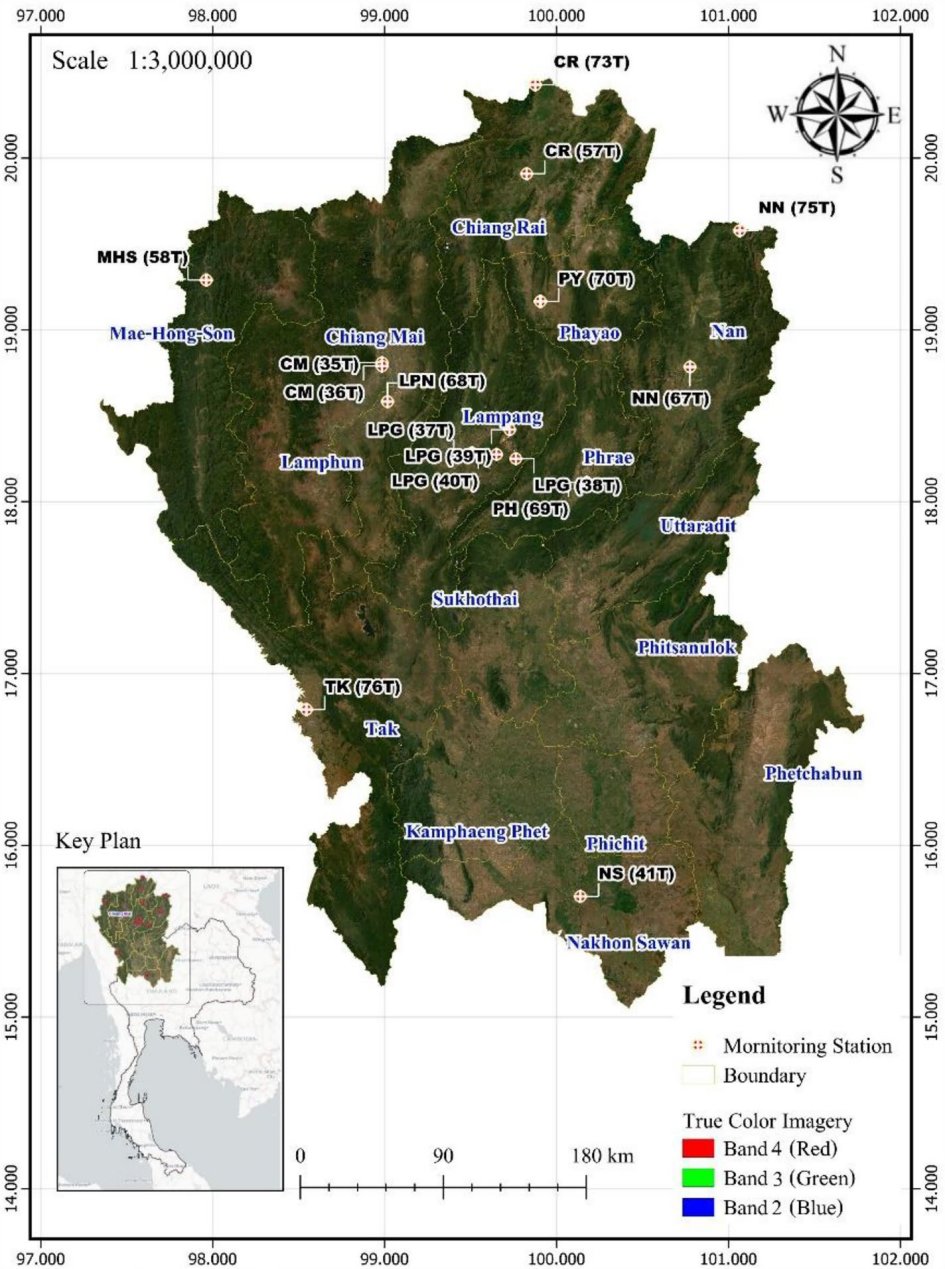
Location coordinates of 16 monitoring stations operating in northern Thailand.

The inventory data for this study included PM_2.5_ and PM_10_ concentrations, represented as annual mean concentrations in μg/m^3^ from the Pollution Control Department (PCD) of Thailand in ten provinces for the period of 2014 to 2018. The locations of monitoring stations are indicated by cross symbols on Landsat-9 images as shown in Fig. 4 and Table 1.

**Table 1:** The monitoring data set on annual mean of PM_2.5_ and PM_10_ concentrations in northern Thailand.

No	Station	Abbreviation	Annual mean PM_2.5_ concentrations (μg/m^3^) (2016–2018)
1	Chiang Mai (35 T)	CNX (35 T)	13.03
2	Chiang Mai (36 T)	CNX (36 T)	25.67
3	Tak (76 T)	TK (76 T)	10.77
4	Nan (75 T)	NN (75 T)	10.85
5	Lampang (40 T)	LPG (40 T)	23.44

No	Station	Abbreviation	Annual mean PM_10_ concentrations (μg/m^3^) (2014–2018)
1	Chiang Rai (57 T)	CR (57 T)	38.27
2	Chiang Rai (73 T)	CR (73 T)	42.25
3	Chiang Mai (35 T)	CNX (35 T)	43.78
4	Chiang Mai (36 T)	CNX (36 T)	45.35
5	Nakhon-Sawan (41 T)	NS (41 T)	50.20
6	Nan (67 T)	NN (67 T)	38.27
7	Phayao (70 T)	PY (70 T)	36.43
8	Phrae (69 T)	PH (69 T)	43.47
9	Mae-Hong-Son (58 T)	MHS (58 T)	38.42
10	Lampang (37 T)	LPG (37 T)	44.45
11	Lampang (38 T)	LPG (38 T)	39.17
12	Lampang (39 T)	LPG (39 T)	33.15
13	Lampang (40 T)	LPG (40 T)	43.10
14	Lamphun (68 T)	LPN (68 T)	36.60

The input parameters for calculating the CF or human health damage are presented in Table 2. The calculation of human health damage was taken into consideration.

**Table 2:** Input parameters for calculating the CF for human health damage^22,25,34^.

Pollutant	Diseases	RR (m^3^/μg)	MR_e,j_ (deaths/year)	YLL (year)	BR (m^3^/year)
PM_2.5_	Lung cancer	1.014	3E−04	517.1	4745
	Cardiopulmonary disease	1.013	2E−03	2,830.3	
PM_10_	Lung cancer	1.27	3E−04	517.1	4745
	Cardiopulmonary disease	1.0005	2E−03	2830.3	

Regarding PM_2.5_, the Relative Risk (RR) value for cardiopulmonary disease is 1.013 per μg/m^3^ which includes, ischemic heart disease, stroke, and respiratory diseases. The RR for lung cancer is 1.014 per μg/m^3^^35^. For PM_10_, the RRs for cardiopulmonary and lung cancer are 1.0005^36^. and 1.27 per μg/m^3^^37^, respectively. This study obtained YLL for four age groups (30–49, 50–59, 60–69, and 70 and older), and the MR per health effect were taken from the World Health Organization^34^. The calculation used the number of adults in northern Thailand aged ≥ 30 years, which amounted to 14,252 persons^38^.

### Human health damage

**Figure 5:**
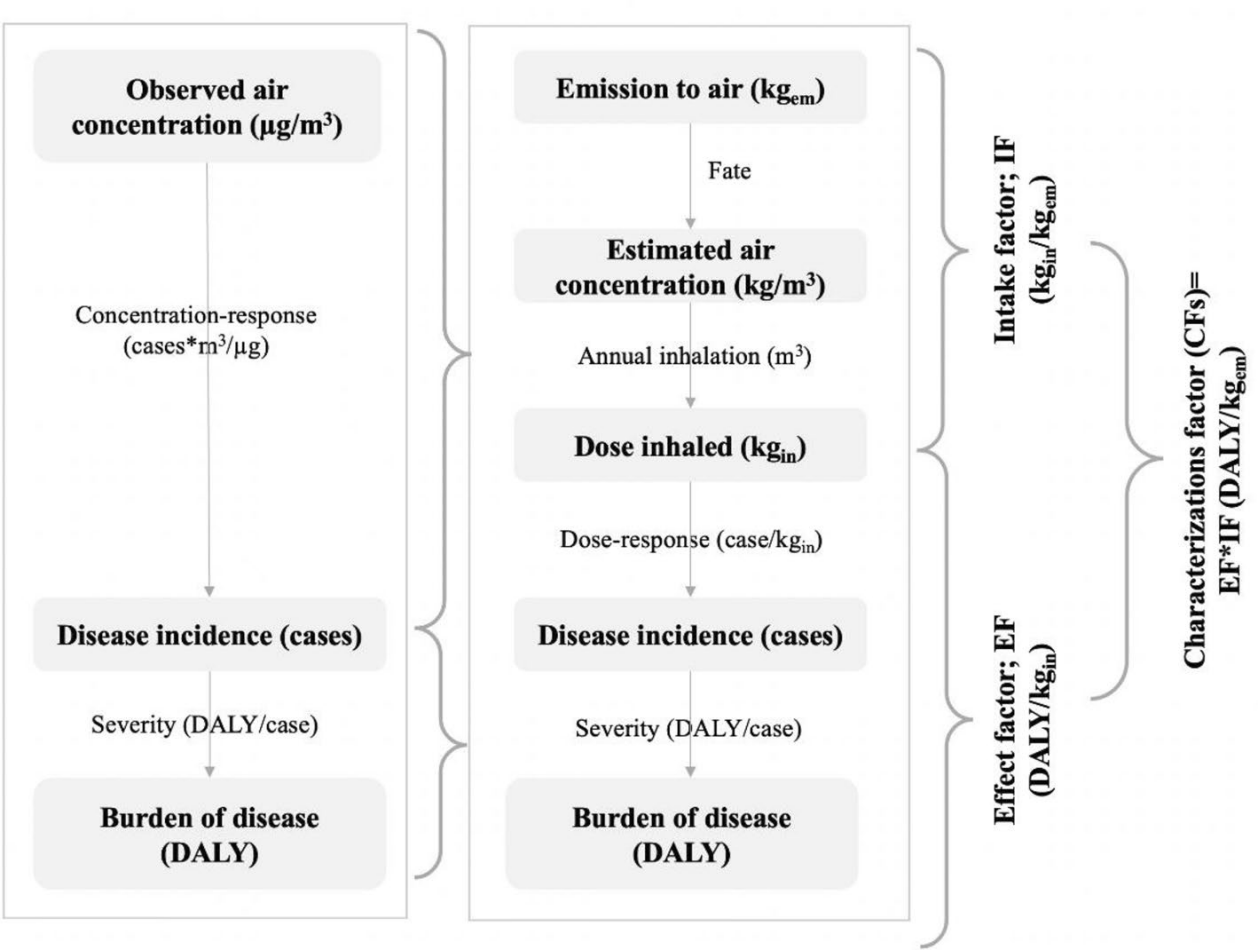
The procedure of determining CF on human health damage.

In this section, the CF for human health damage caused by PM_2.5_ and PM_10_ in 10 provinces of northern Thailand is evaluated. CFs are addressed based on the marginal change in DALYs of humans resulting from a marginal change in the annual concentrations of PM_2.5_ and PM_10_. The applied fate and exposure analysis model is presented in Fig. 5.

#### CFs for human health damage

The data of PM_2.5_ and PM_10_ concentrations, collected from PCD, ranged from the period from 2014 to 2018. This annual dataset allows for the quantification of the burden of disease from PM_2.5_ and PM_10_ related mortality and morbidity due to exposure to these pollutants through Life Cycle Impact Assessment. The calculations and assumptions for CF for human health damage consist of three factors: (a) intake factor (IF), (b) human effect factor (EF) and (c) damage factor (DF). Overall, CF (DALY/kg_emitted_) for human health damage caused by PM_2.5_ and PM_10_ are provided in Eq. (1) below^22,25^:

1 $${CF}_{HH,k,x,i }={\sum }_{j}\left\{({IF}_{k,x,i})\cdot \sum ({EF}_{e,k,j}\cdot {DF}_{e,k,j})\right\}$$

where *IF* is the intake factor, indicating the amount of pollutant inhaled by the population per unit of pollutant emitted (kg_inhaled_/kg_emitted_), *EF* is the human effect factor, which reflects the change in disease incidence resulting from a change in exposure to the pollutant (DALYs/kg_inhaled_), *DF* is the damage factor, which quantifies the harm caused by the pollutant. *CF* is the characterization factor, indicating the annual marginal change in DALYs per unit increase in the ambient concentrations (DALY/substance_emitted_)

#### Intake factor (IF)

Instead of using the more commonly used intake fraction, this study employs an intake factor, *IF*_*pop,x*_ having dimensionless units to expresses the population intake of pollutant *k* (*I*_*pop,k*_ in kg/year) per unit emission of substance *x*^39^. The reason for this choice is that the intake fraction would imply the intake of a fraction of the emission itself, whereas in the case of secondary aerosols, the primary emission is a different substance.

The calculation of *IF*_*pop,x*_ can be performed using the formula described by Kassomenos et al.^22^

2 $${IF}_{pop,x}=\frac{{dI}_{pop,m}}{{dM}_{x}}=\left(IH\cdot N\right)\frac{{dC}_{m}}{{dM}_{x}}$$

where *N* is the number of inhabitants in the area, *C*_*m*_ is the annual average concentration of the pollutant, *m* (kg/m^3^), *M*_*x*_ is the annual emissions of substance *x* (kg/year), *IH* is the average human health intake rate (13 m^3^/day = 4745 m^3^/year, US EPA 1997).

For this study, it is assumed that the CF values were calculated per person (i.e., N=1). Additionally, concentration increments of 1% were used, i.e., dC/dM (the change in concentration per unit change in emissions, *M*_*x*_) was considered as a 1% increase of the average concentration *C*_*m*_.

#### Effect factor (EF)

An effect factor (EF) represents the human effect caused by the pollutant in the receptor and quantifies the change in disease incidence resulting from a change in exposure. It is determined by dividing the concentration-response function (*CRF)* in m^3^/year/kg by the *BR* (m^3^/year)

3 $${EF}_{e,k,j}=\frac{{dINC}_{k,j}}{{dEXP}_{k,j}}=\frac{{CRF}_{e,k,j}}{BR}$$

The region-specific *CRF* can be calculated using the following Equation^25^:

4 $${CRF}_{e,k,j}=\frac{\left({RR}_{e,k}-1\right)\cdot {MR}_{e,j}}{\left({RR}_{e,k}-1\right)\cdot {C}_{k,j}+1}$$

where *CRF* is the concentration–response function (m^3^/year/kg), *RR*_*e,k*_ is the relative risk associated with obtaining a specific health effect, *e*, due to exposure to the pollutant, *k* (per μg/m^3^), *MR*_*e,j*_ is the mortality rate for the specific health effect, *e*, in region *j* in terms of deaths/person/year, *C*_*k,j*_ is the yearly average background concentration of pollutant k in in region (μg/m^3^), *BR* is the breathing rate (m^3^/year).

By applying these calculations, the specific CRF values can be obtained, which quantify the relationship between pollutant exposure and health effects in a given region.

#### *Damage factor*^25^*(DF)*

A damage factor (*DF*_*e*,*k*,*j*_) is a measure of the years of life lost (YLL) associated with a specific health effect (*e*) per incidence case. The YLL value associated with the health effect (*e*) per incidence case in region *j* for a specific pollutant *k* is estimated using data from the from the World Health Organization^34^. The DF can be calculated using the following equation:

5 $${DF}_{e,k,j}=\frac{{dYLL}_{e,k,j}}{{dINC}_{e,k,j}}$$

This equation allows for the quantification of the impact of a specific health effect caused by a pollutant in a given region in terms of years of life lost.

## Results and discussion

### Human health damage

#### Results of IF

The results of the intake factor (IF) analysis are presented in Table 3. The findings showed that the highest IF value for inhaled PM_2.5_ occurred at Chiang Mai, CNX (36T), station, which is situated in a high traffic area, with a value of approximately 1.22E−06. This indicates that the source of PM_2.5_ at the CNX (36T) station contributed to a relatively higher PM_2.5_ concentration compared to other stations in different provinces. Additionally, Chiang Mai, where the CNX (36T) station is located, has a high population denstity. As a result, Chiang Mai had the highest IF value for PM_2.5_, followed by Lampang, LPG (40T), another station in Chiang Mai, CNX (35T) and Nan, NN (75T), stations, respectively.

**Table 3:** Results of IF.

No	Station	IF
PM_2.5_ monitoring stations		
1	CNX (36 T)	1.22E−06
2	LPG (40 T)	1.11E−06
3	CNX (35 T)	6.18E−07
4	NN (75 T)	5.15E−07
5	TK (76 T)	5.11E−07
PM_10_ monitoring stations		
1	NS (41 T)	2.38E−06
2	CNX (36 T)	2.15E−06
3	LPG (37 T)	2.11E−06
4	CNX (35 T)	2.08E−06
5	PH (69 T)	2.06E−06
6	LPG (40 T)	2.05E−06
7	CR (73 T)	2.00E−06
8	LPG (38 T)	1.86E−06
9	CR (57 T)	1.82E−06
10	NN (67 T)	1.82E−06
11	MHS (58 T)	1.82E−06
12	LPN (68 T)	1.74E−06
13	PY (70 T)	1.73E−06
14	LPG (39 T)	1.57E−06

On the other hand, the highest IF value for inhaled PM_10_ was observed at Nakhon-Sawan, NS (41T) station. Nakhon-Sawan is a large city that encompasses both urban and agricultural areas. The station at Nakhon-Sawan only measured PM_10_ and did not have PM_2.5_ measurements during the study period. The region is predominantly dedicated to agriculture, with rice and maize crops being the most common. Nakhon-Sawan also has a high population density. Based on the available PM_10_ monitoring data, the results indicated that Nakhon-Sawan had the highest IF value for PM_10_, followed by Chiang Mai, CNX (36T), and Lampang, LPG (37T), stations, respectively.

These findings suggest that the concentration and distribution of PM_2.5_ and PM_10_ pollutants, as well as population density and specific local factors such as traffic and agricultural activities, contribute to variations in the IF values across different stations and provinces in northern Thailand.

#### Results of EF

The results of the effect factor (EF) analysis, expressed as EF factors, are presented in Table 4. The focus of this study was on EF for mortality associated with dominant diseases, namely lung cancer disease and cardiopulmonary diseases, which included ischemic heart disease, stroke, and respiratory diseases^34^, caused by PM_2.5_ and PM_10_ pollutants.

**Table 4:** Results of EF for mortality due to PM_2.5_, PM_10_ exposure in Northern Thailand.

No	Station	EF of lung cancer (DALYs/kg_inhale_)	EF of cardiopulmonary (DALYs/kg_inhale)_
PM_2.5_ monitoring stations			
1	TK (76 T)	7.86E−10	5.03E−09
2	NN (75 T)	7.85E−10	5.02E−09
3	CNX (35 T)	7.64E−10	4.90E−09
4	LPG (40 T)	6.81E−10	4.39E−09
5	CNX (36 T)	6.65E−10	4.30E−09
PM_10_ monitoring stations			
1	LPG (39 T)	1.75E−09	2.17E−10
2	PY (70 T)	1.61E−09	2.16E−10
3	LPN (68 T)	1.60E−09	2.16E−10
4	CR (57 T)	1.54E−09	2.16E−10
5	NN (67 T)	1.54E−09	2.16E−10
6	MHS (58 T)	1.53E−09	2.16E−10
7	LPG (38 T)	1.51E−09	2.16E−10
8	CR (73 T)	1.41E−09	2.16E−10
9	LPG (40 T)	1.38E−09	2.16E−10
10	PH (69 T)	1.37E−09	2.16E−10
11	CNX (35 T)	1.36E−09	2.16E−10
12	LPG (37 T)	1.34E−09	2.16E−10
13	CNX (36 T)	1.32E−09	2.16E−10
14	NS (41 T)	1.20E−09	2.15E−10

The variables used in the calculation of EF include the concentration–response function (CRF), relative risk (RR), mortality rate (MR), annual average background concentration of the pollutant, and breathing rate (BR). With the exception of CRF, which was related to the annual average background concentration of the pollutant at each station, baseline parameters were mostly used for the calculations. These variables can influence the EF value of each specific area.

The highest EF value for PM_2.5_-related mortality from lung cancer and cardiopulmonary diseases was found at the Tak, TK (76 T), station, with values of approximately 7.86E-10 DALYs/kg_inhaled_ and 5.03E−09 DALYs/kg_inhaled_, respectively. It is worth noting that Tak, where the TK (76 T) station is located, is in close proximity to neighboring countries like Myanmar, and the increased concentration observed in this area from 2016 to 2018 could be influenced by transboundary pollution.

For PM_10_ exposure, the highest EF values for lung cancer and cardiopulmonary diseases were observed at the Lampang, LPG (39 T), station, which is situated in the coal power plant area in the Mae-Moh district of Lampang. The calculated EF values for lung cancer and cardiopulmonary diseases at this station were approximately 1.75E−09 DALYs/kg_inhaled_ and 2.17E−10 DALYs/kg_inhaled_, respectively.

These results indicate the varying EF values for different diseases and pollutants across the monitoring stations in northern Thailand. Specific local factors, such as transboundary pollution and proximity to coal power plant areas, can contribute to higher EF values in certain regions. 

#### Results of CFs

**Figure 6:**
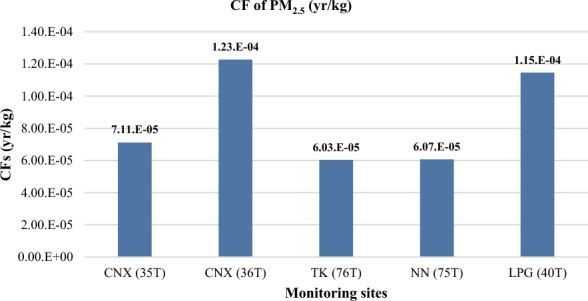
Results of CF for PM_2.5_.

**Figure 7:**
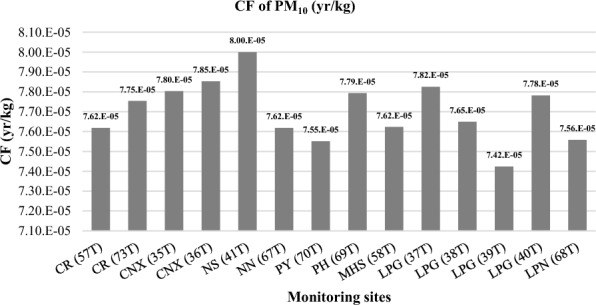
Results of CF for PM_10_.

The results of the characterization factor (CF) calculation for human health damage, which represents the marginal change in DALYs (Disability-Adjusted Life Years) due to a marginal change in the annual concentrations of PM_2.5_ and PM_10_, are shown in Figs. 6 and 7. The highest CF value for PM_2.5_ was found at the Chiang Mai, CNX (36T), located in the traffic area of Chiang Mai city at the Yupparaj Wittayalai School, with a value of approximately 1.23E−04 year/kg. The next highest CF value was observed at the Lampang, LPG (40T), station, situated in the power plant area of Lampang, at approximately 1.15E−04 year/kg. These results correspond to the intake factor (IF) values of PM_2.5_, indicating that the most affected areas in terms of health damage from PM_2.5_ are Chiang Mai and Lampang provinces.

Regarding the CF of PM_10_, the results showed that the highest CF values were observed at Nakhon-Sawan, NS (41T), and Chiang Mai, CNX (36T), stations with values of 8.00E−05 year/kg and 7.85E−05 year/kg, respectively. These CF results align with the IF values of PM_10_, which are highest in the urban areas of Nakhon-Sawan and Chiang Mai. It is worth noting that the CF of PM_2.5_ appears to be higher than that ofPM_10_, indicating that particulates with an aerodynamic diameter less than or equal to 2.5 micrometers have a greater impact on human health.

In addition, this study calculated the average years of life lived with disability (YLD) associated with ambient PM_2.5_ in northern Thailand, which amounted to approximately 48,372 years per 100,000 people. This calculation was based on the PM_2.5_ emissions data for Thailand in 2018 from the Emissions Database for Global Atmospheric Research (EDGAR)^40^. Furthermore, it was found that the number of deaths from lung cancer and cardiopulmonary diseases caused by PM_2.5_ was approximately 0.04% and 0.06% of the population of northern Thailand, respectively. Similarly, for PM_10_ emissions in Thailand in 2018, the YLD for the entire population of northern Thailand was estimated to be around 59,064 years. The percentage of deaths from lung cancer and cardiopulmonary diseases caused by PM_10_ was approximately 0.06% and 0.08%, respectively.

#### Comparison with other studies

The results of this study were compared to those obtained from published Life Cycle Assessments (LCAs) that focused on characterization factors (CFs) for human health damage^19,22,23,25^. The intake factor (IF) values for PM_2.5_ in this study ranged from 5.1.E−07 to 1.2.E−06, whereas Van Zelm et al.^25^ reported a value of 1.94E−06 for primary PM_2.5_ for the entire country of Thailand.

**Figure 8:**
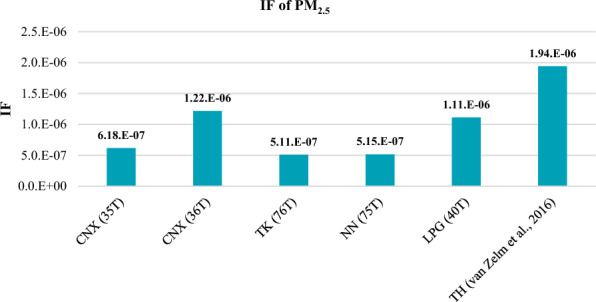
Comparison of IF for PM_2.5_ with another study.

It is worth noting that the IF values obtained in this study for the five PM_2.5_ monitoring stations in the northern region were lower than the IF value reported by Van Zelm et al. for the entire country. This difference can be attributed to the fact that the sources of PM_2.5_ in northern Thailand, such as agricultural waste burning or forest fires, may be different from other regions in Thailand. Therefore, the IF values for PM_2.5_ in the northern region may not be representative of the entire country. This comparison in illustrated in Fig. 8, which presents the IF values for PM_2.5_ from this study along with values from other studies.

When comparing the characterization factor (CF) for PM_2.5_ in this study with the regionalized CF by Van Zelm et al. for the entire country of Thailand, it was found that the CF in this study is lower. Van Zelm et al. study aimed to quantify the overall human health damage caused by a specific pollutant per unit of emission of a primary PM_2.5_ precursor across Thailand. Therefore, the human health damage for the five PM_2.5_ stations in northern Thailand is lower than that for the entire country.

**Figure 9:**
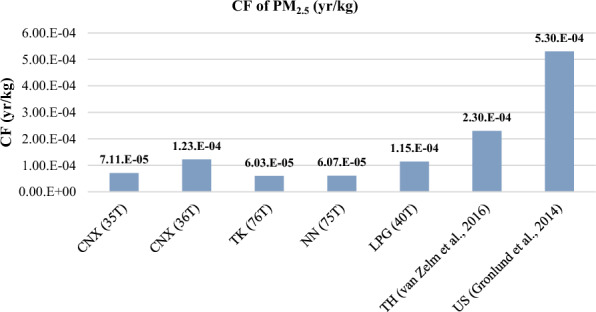
Comparison of CF for PM_2.5_ with other studies.

Furthermore, the average results of this study were compared to CF values from 63 densely populated urban areas in the United States^23^. It was found that the CF values in this study are lower than those for the US metropolitan areas. The differences can be attributed to variations in emissions sources and the characteristics of a large country like the United States, which includes urban, rural, and remote locations. Additionally, the input data used for CF calculations, such as concentration and dose–response factors, annual mortality rate, and specific diseases in each metropolitan area, influenced the CF values. This comparison is depicted in Fig. 9, which presents the CF values for PM_2.5_ from this study along with values from other studies.

**Figure 10:**
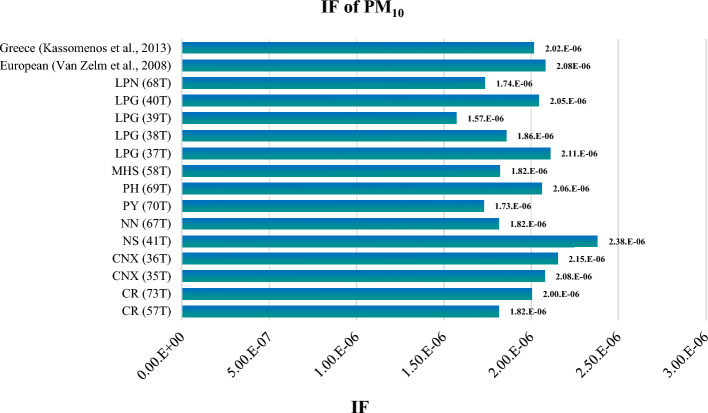
Comparison of IF for PM_10_ with other study.

**Figure 11:**
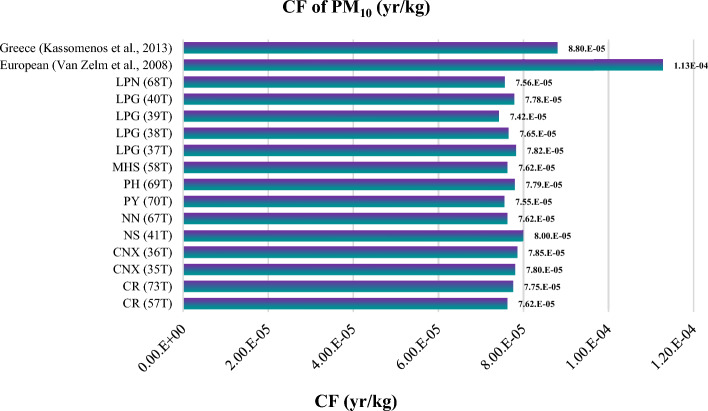
Comparison of CF for PM_10_ with other studies.

The comparison results of the intake factor (IF) and characterization factor (CF) for PM_10_ with other studies are presented in Figs. 10 and 11. When comparing the IF values for PM_10_ from this study with studies conducted in Europe^19^ and greater area of Athens, Greece^22^, it was found that the IF values for all 14 stations in this study are lower. This indicates that the population intake of PM_10_ per unit of emission of substance is lower in northern Thailand compared to Europe and Athens. The differences in IF values can be attributed to variations in emission sources and concentrations between countries. It suggests that the PM_10_ emissions released in the northern region of Thailand may be lower compared to other countries in Europe and Greece.

However, when comparing the IF values for each station in northern Thailand to the literature values, it is observed that some stations, such as Nakhon-Sawan, NS (41 T), exhibit higher IF values. This indicates that these stations have higher concentrations of PM_10_, leading to higher population intake per unit of emission. This can be attributed to specific local factors and sources in those areas.

In summary, the IF and CF values for PM_10_ in northern Thailand, when compared to other studies, show lower values on a broader regional scale (Europe and Athens), but there are variations within the region itself.

### Summary

The differences observed in the CF values for both PM_2.5_ and PM_10_ between this study and other studies can be attributed to various factors related to emissions sources, meteorology, climate, geography, and other contextual variables. Each country has its own unique mix of air pollutants emitted, which can be influenced by industrial activities, transportation, energy production, and other sources.

Additionally, meteorological conditions such as wind patterns, temperature, and precipitation can affect the dispersion and transport of air pollutants, leading to variations in concentration levels. Climate factors such as temperature inversions or stable atmospheric conditions can also contribute to higher pollutant concentrations in specific areas.

Geographical factors play a role as well. Countries and regions with different topography, land use patterns, and proximity to pollution sources may experience varying levels of pollution and associated health impacts. Urban areas, with their higher population densities and increased pollution sources, may have different CF values compared to rural or remote areas.

Furthermore, CF calculations are influenced by other factors such as mortality rates, years of life lost, and incident cases, which can vary from country to country. These factors reflect the specific health burden and vulnerability of the population in each location, and they contribute to the estimation of CF and the overall health damage associated with air pollution.

To ensure accurate and precise estimation of health damages specific to each country, it is crucial to use appropriate and context-specific input parameters for CF calculations. This includes considering the unique emissions sources, meteorological conditions, climate patterns, geography, and health-related factors of the particular country or region under study.

With regards to the results of this study, we found that there is some limitation of these input parameters for calculating the CF for human health damage that were used. Due to the lack of observed data, values from scientific reports, existing research, and publications were referred to. Only the results of IF and CF in Europe and Thailand are compared. Considering the IF and CF estimated of pollutant in northern Thailand for this study, the results of the fate and exposure analysis were significant. It is important to observe and analyze how modeled concentrations and temporal variations of emission substances in different regions are represented. This study was limited due to a lack of observed data for calculating the EF of human caused by the pollutant, which represents the change in disease incidence due to a change in exposure as a result of the estimated EF. More specifically, the value of the region-specific CRF is necessary for accurate calculation. Moreover, the DF calculation used statistical data of YLL associated with the health effect, which was estimated per region. Therefore, more observed data is required to estimate the result of the diseases on which we have focused. In summary, the combined intake, effect, and DF presented in CFs can reveal differences in area depending on the context that may influence PM characterization for different areas. In the long term, more specific data for CF calculation is required for related variables such as RR value, MR, or YLL in each area in order to evaluate the most accurate and consistent health damage and implement the results to benefit proper practice/policy/action in terms of air pollution management.

## Conclusions

The severity of the environmental impacts due to the increased amount of PM_2.5_ and PM_10_ in the ambient air in terms of the annual marginal change in the DALYs due to a marginal increase in the ambient concentrations in the northern Thailand region were determined. According to the study results, the annual average number of years of life lived with disability due to ambient PM_2.5_ in northern Thailand is approximately 41,372 years, while PM_10_ is approximately 59,064 years. We found that there were some limitations in the input data used for the calculations; these data limitations were met by using values from the scientific literature. To determine the actual severity of human health damage due to air pollution, the proper and precise input parameters should be utilized. Overall, the findings and insights from this research have the potential to drive positive change and contribute to the development of more effective recommendations and policies that address the critical need for mitigating air pollution while taking on climate change.

## Recommendations

Further research needs to be done to emphasize more accurate results. We plan to develop more detailed, updated, and accurate input data which refer to specific diseases, MR, YLL and RR to get a better and accurate model of human health damage. We could determine the actual severity of air pollution’s impact on human health in terms of the number of years of life lived with disability because of PM_2.5_ and PM_10_ in northern Thailand. We hope that the current findings will help to inform future research on this important topic related to years of life loss of pollutant emitted.

Based on our research results, which indicate a link between air pollution and its impact on air quality, it is critical to adopt new rules and strategies to reduce air pollution. These regulations and policies can play a significant role in improving air quality and mitigating the harmful effects of pollution on human health and the environment. By considering emerging regulations and policies, we can ensure that our efforts to reduce air pollution align with the evolving landscape of environmental policies and contribute to long-term improvements in air quality. As we have known, currently air pollution decreases the average Thai resident’s life expectancy by 1.8 years compared to what would be possible if the WHO guideline was met permanently. However, air pollution levels in the Northern region are 18 to 52 percent higher than the national average mentioned by Thailand Clean Air Network. This could lead to more issues on the Air Quality Life Index (AQLI) which is a pollution index that measures the impact of particulate air pollution on life expectancy developed by the Energy Policy Institute at the University of Chicago. The index also illustrates how air pollution policies can increase life expectancy when they meet the World Health Organization’s guideline for what is considered safe. Following this idea, recently the PCD’s proposed plan to enforce the air quality safety level by lowering the acceptable standard of PM_2.5_ in the atmosphere from an average of 50 μg/m^3^ per 24 h to 37.5 μg/m^3^ per 24 h was approved by the National Environmental Board (The new regulation will take effect on June 1, 2023)^41^.

This could be an effort to protect and improve national air quality by regulating emissions from mobile and stationary sources, such as reducing agricultural waste burning, supporting biomass management and forest fire management, transportation management, and public awareness through the use of air quality monitoring stations, and providing more awareness on the health effects of air pollution. Despite the fact that we have an existing national policy for air quality, it is still necessary and urgently need to better establish the appropriate air quality guidelines/policy for northern Thailand.

## Data Availability

The datasets analyzed during the current study are available in the PCD of Thailand repository, [http://air4thai.pcd.go.th/].

## Abbreviations

PM_2.5_: Particles less than 2.5 µm in diameter
PM_10_: Particles less than 10 µm in diameter
CR: Chiang Rai province
CNX: Chiang Mai province
TK: Tak province
NS: Nakhon-Sawan province
NN: Nan province
PY: Phayao province
PH: Phrae province
MHS: Mae-Hong-Son province
LPG: Lampang province
LPN: Lamphun province
RR: Relative risk
MR: Mortality rate
YLL: Years of life lost
BR: Breathing rate
CFs: Characterization factors
IF: Intake factor
EF: Effect factor
DF: Damage factor
CRF: Concentration-response function
DALYs: Disability adjusted life years
LCA: Life cycle assessment
PCD: Pollution control department
WHO: World Health Organization
