# Author Correction: Association between ambient air particulate matter and human health impacts in northern Thailand

**DOI:** 10.1038/s41598-023-42770-2

**Published:** 2023-09-18

**Authors:** Titaporn Supasri, Shabbir H. Gheewala, Ronald Macatangay, Anurak Chakpor, Surat Sedpho

**Affiliations:** 1 Atmospheric Research Unit, National Astronomical Research Institute of Thailand, Chiang Mai, Thailand; 2 Energy Engineering Program, Faculty of Engineering, Chiang Mai University, Chiang Mai, Thailand; 3 The Joint Graduate School of Energy and Environment (JGSEE), King Mongkut’s University of Technology Thonburi, Bangkok, Thailand; 4 Center of Excellence on Energy Technology and Environment, Ministry of Higher Education, Science, Research and Innovation, Bangkok, Thailand; 5 Institute of Environmental Science and Meteorology, University of the Philippines Diliman, Quezon City, Philippines; 6 School of Energy and Environment, University of Phayao, Phayao, Thailand

Correction to: *Scientific Reports* https://doi.org/10.1038/s41598-023-39930-9, published online 07 August 2023

In the original version of this Article, Titaporn Supasri was omitted as a corresponding author. Correspondence and requests for materials should also be addressed to titaporn@narit.or.th

The original Article has been corrected.

